# Review of post bariatric surgery effects on common genitourinary physiology

**DOI:** 10.1590/S1677-5538.IBJU.2017.0416

**Published:** 2018

**Authors:** Aline A. Yacoubian, Rami Nasr

**Affiliations:** 1 Division of Urology, Department of Surgery, American University of Beirut Medical Center, Beirut, Lebanon

**Keywords:** Obesity, Bariatric Surgery, Nephrolithiasis, Fertility

## Abstract

*Background:* Obesity is a worldwide challenging health problem. Weight loss through medical management of obesity has not always been successful, thus, giving rise to the need for surgical intervention. Bariatric surgery has been shown to be helpful for morbidly obese patients. However, studies have also shown the effect of surgery on stone formation, fertility and erectile function. This review summarizes the main findings of several studies that analyze stone formation and fertility in men as well as erectile function post bariatric surgery. The underlying pathophysiologic alterations post bariatric surgery include increased absorption of oxalate leading to hyperoxaluria, hypocitraturia and increased urinary calcium oxalate supersaturation. Contradicting data exist on the effect of bariatric surgery on fertility and erectile function. Further studies are needed to analyze the mechanisms.

## INTRODUCTION

Obesity is a public health concern with increased prevalence in the past two decades (1). The highest peak is in women and men aged between 20 and 40 years (2). In adults aged 20 years and above, it is defined as body mass index (BMI) equal to or greater than 30 kg/m^2^ (3). Obesity affects more than one third of adults in the United States (US) (4). The 2011-2012 National Health and Nutrition Examination Survey (NHANES) conducted on 9120 individuals in the US showed that 31.8% of the 584 youth (youth defined as those from birth until two years of age) were either overweight or obese, while 16.9% of the youth were obese (3). Obesity causes several health risks such as diabetes, hypertension, dyslipidemia, cardiovascular disease, sleep apnea, etc. (5). It can even affect reproductive functions in both sexes and lead to pregnancy/perinatal or offspring adverse effects (1). In men, it may cause oligozoospermia and asthenozoospermia (1), erectile dysfunction (2) and subfertility (6). The estimated annual medical costs of illness pertinent to adult obesity exceed 200 US billion (5). This implies that 20.6% of the US national health expenditures are spent on obesity-linked illnesses (5).

Bariatric surgery is an alternative for patients with BMI ≥40 kg/m^2^ or BMI ≥35 kg/m^2^ and who suffer from coexisting morbidities linked to obesity (6). The currently performed bariatric surgeries are either restrictive such as sleeve gastrectomy (SG) and laparoscopic adjustable gastric band (LAGB) or combined restrictive/mal-absorptive like Roux-en-Y gastric bypass (RYGB) (1). In 2011, around 340,000 bariatric surgeries were performed across the globe and RYGB was the most common procedure (47%), followed by SG (28%) and LAGB (18%) (1). The number of bariatric surgeries rose to 468,609 in 2013 worldwide (7) and 196,000 procedures were performed in the US alone in 2015 (8). Although bariatric surgery can lead to weight reduction and decrease of the above mentioned health problems, it leads to numerous physiological alterations that are not just restricted to the gastrointestinal tract (4). Clinical data propose that bariatric surgery is linked to nephrolithiasis due to variations in urinary volume, oxalate and citrate (9). However, other studies show conflicting effect on fertility in men and erectile function (10, 11).

## OBJECTIVES

This review addresses the physiological alterations of bariatric surgery on the genitourinary system.

## MATERIALS AND METHODS

We comprehensively searched the databases of Pubmed on March 21 and 22 and August 31, 2017 for clinical trials, review papers and meta-analyses focusing on the effect of bariatric surgery on the incidence of stone formation, pathophysiology of stone risk and fertility in men as well as erectile function. The inclusion criteria were based on the most relevant, most recent and most cited studies about clinical and experimental literature that discuss pathophysiology of stone risk and mechanism of sexual and erectile dysfunction in men after bariatric surgery. A summary table of studies about changes in genitourinary system, fertility and sexual function in humans after bariatric surgery is presented in Table-1 and of animal studies in Table-2 and arranged by type of study (retrospective, prospective, cross-sectional and case reports respectively).

**Table 1:** Human studies about physiological alterations of bariatric surgery on the genitourinary system arranged by study type.

Author	Year	Study type	Surgery type	Sample size	Findings
Nelson et al. (14)	2005	Retrospective study	RYGB	23 patients	Calcium oxalate nephrolithiasis in 21 patients. Enteric hyperoxaluria with oxalate nephropathy in 2 patients. Increased 24-hour excretion of urinary oxalate and calcium oxalate supersaturation.
Matlaga et al. (13)	2009	Prospective study	RYGB	4693 patients vs. 4693 controls	In operated group: Increased risk of kidney stone.
Park et al. (15)	2009	Prospective study	RYGB	45 patients	Increased urinary oxalate and calcium oxalate supersaturation. Decreased urinary citrate and total urinary volume.
Penniston et al. (20)	2009	Prospective study	27 RYGB 12 gastric binding	39 patients	Decreased urinary volume in RYGB and gastric banding. Decreased urinary calcium in RYGB. Decreased urinary citrate in 14 RYGB patients and 1 gastric binding patient. Increased urinary oxalate in RYGB.
Dufey et al. (16)	2010	Prospective study	RYGB	21 patients	Increased urinary oxalate excretion. *De novo* hyperoxaluria. Increased hypocitraturia.
Reis et al. (24)	2010	Prospective study	Gastric bypass	20 patients 20 cohorts	In operated group: Increased International Index of Erectile Function score, total testosterone, and follicle - stimulating hormones. Decreased prolactin level.
Kumar et al. (17)	2011	Prospective study	9 RYGB 2 biliopancreatic diversion-duodenal switch	11 patients	Increased urine oxalate excretion. Increased plasma oxalate and urine calcium oxalate supersaturation. Increased fecal fat excretion. Decreased total urine volume.
Valezi et al. (19)	2013	Prospective study	RYGB	151 patients	Increased urinary oxalate. Increased urinary uric acid. Decreased urinary volume.
Rosenblatt et al. (28)	2013	Prospective study	RYGB	23 patients 14 obese controls 14 lean controls	In operated group: Improved erectile dysfunction and overall satisfaction.
Agrawal et al. (18)	2014	Prospective study	RYGB	11 patients	Increased urinary oxalate. Decreased citrate. Decreased urine volume.
Lieske et al. (12)	2015	Prospective study	591 RYGB 105 mal-absorptive procedure 56 restrictive 7 other	762 patients vs. 759 controls	In operated group: Risk of stones.
Sarwer et al. (25)	2015	Prospective study	RYGB	32 patients	Increased total testosterone and sex hormone binding globulin.
Goiten et al. (26)	2015	Prospective study	36 laparoscopic sleeve gastrectomy 12 RYGB	48 patients	Improved general satisfaction, desire and erection.
El Bardisi et al. (27)	2016	Prospective study	SG	46 patients	Increased serum testosterone.
Samavat et al. (23)	2017	Prospective study	23 RYGB	23 patients 8 controls	In operated group: Increased gonadotropins, total testosterone, sex-hormonebinding-globulin and calculated free testosterone. Decreased estradiol. Increased semen volume and viability. Improved progression and total motility and total sperm number. In both groups: Worsened sperm morphology.
Maalouf et al. (22)	2010	Cross-sectional study	RYGB	19 patients 19 controls	In operated group: Increased urine oxalate. Decreased urine citrate. Decreased urine calcium.
di Frega et al. (11)	2005	Case reports	RYGB	6 patients	Non-obstructive azoospermia. Spermatogenesis arrest.
Sermondade et al. (10)	2012	Case reports	1 SG 2 RYGB	3 patients	Extreme oligoasthenoteratozoospermia. No azoospermia.

**Table 2:** Animal studies about changes in urinary profile after bariatric surgery.

Author	Year	Study type	Surgery type	Sample size	Findings
Canales et al. (21)	2013	Prospective study	RYGB	19 rats 16 controls	In operated group: Increased urinary volume. Increased urine pH. Increased urine oxalate excretion.
Choi et al. (29)	2014	Prospective study	Gastric bypass	15 subject rats 10 control rats	In operated group: Increased intracavernous pressure/mean arterial pressure ratio.
					Increased cavernosum smooth muscle/collagen ratio. Increased endothelial nitric oxide synthase and neuronal nitric oxide synthase.
					Decreased expression of Rho kinase and of 8-hydroxy-2-deoxyguanosine levels.

## RESULTS & DISCUSSION

### Bariatric surgery and nephrolithiasis

Bariatric surgery causes higher incidence of nephrolithiasis (7, 8, 12). The risk of kidney stone formation after RYGB has been studied over the last decade (9). One study reported that the incidence of stone among 4639 patients post RYGB was 7.65% vs. 4.63% of obese controls (p<0.0001) (13). The person-time stone incidence was projected to be 16.62 stones per 1000 person-years for RYGB (13). A meta-analysis of four articles (1 randomized trial and 3 cohort studies) showed that the incidence of kidney stone formation depends on the type of procedure (7). Patients who have undergone RYGB had an overall increased risk of 1.73 vs. those who have not undergone any procedure, while patients who have undergone laparoscopic banding or SG had a decreased risk of 0.37 (7). In a population-based study with 762 comorbidity-matched patients vs. 759 controls, the incidence of stone formation was more common in patients who underwent bariatric surgery vs. controls (p<0.01) (12). The risk was highest among those who underwent mal-absorptive procedures, followed by RYGB and restrictive procedures (12).

The mechanism for nephrolithiasis post bariatric surgery is complex, but involves different pathologies such as hyperoxaluria, hypocitraturia and aciduria (8). Hyperoxaluria is common in patients who have undergone RYGB because of changes in intestinal microbial flora that lead to increased oxalate absorption (8). The relationship between hyperoxaluria and stone formation was firstly investigated by Nelson et al. in a study on 23 patients (14 men and 9 women; mean age = 45 years) (14). There were 21 patients who developed nephrolithiasis and 2 developed oxalate nephropathy (14). Prospective studies on non-stone formers showed hyperoxaluria in 45 patients (15), doubling of urinary oxalate excretion in 21 patients (16), increase in urine oxalate and decreased urine volume in 11 patients (17) and increased urine oxalate in 13 patients after patients underwent RYGB (18). Another study among 151 patients followed up for a year after RYGB showed significant higher urinary oxalate (24 vs. 41 mg; p<0.001) and urinary uric acid (545 vs. 645 mg; p<0.001) (preoperative vs. postoperative, respectively) (19). A retrospective study on 39 non-stone formers (28 females and 11 males, mean age = 51.2 years) showed that urinary oxalate excretion was about 150% higher in the RYGB group vs. the gastric band group (20).

Calcium oxalate stones are more common after bariatric surgery. In a comorbidity-matched study of 762 patients who underwent surgery vs. 759 controls (mean age = 45 years), patients with history of stone were more likely to form stones after surgery (p<0.001) (12). Urine oxalate excretion increased after bariatric surgery (<8 months vs. >8 months, p<0.001) in 55 patients with follow-up stones, in 248 patients without follow-up stones and in 20 obese controls with follow-up stones (12). Moreover, mal-absorptive bariatric surgery causes hyperoxaluria by the absorption of fatty acids and/or bile salts followed by the saponification of calcium ions and fat-soluble vitamins (9). This saponification leads to decreased calcium in the intestinal lumen, decreased calcium-oxalate as well as increased free oxalate in the small and large intestine (9). By this way, dietary oxalate becomes available for absorption by the gut and excretion by the kidneys (20). Another possible mechanism for the cause of hyperoxaluria is the colonization of bowel bacteria called *Oxalobacter formigenes* that metabolize intestinal oxalate after surgery (19). In an animal study, 16 diet induced obese rats underwent sham surgery as controls and 19 RYGB rats were introduced to a normal calcium, high fat (40%) diet (with or without 1.5% potassium oxalate) for five weeks and then given a normal (10%) fat diet for two weeks (21). RYGB rats had eightfold higher fecal fat excretion (p<0.001), heavier stools (p=0.02) and fivefold increase in urine oxalate excretion (p<0.001) (21).

Another reason for nephrolithiasis is hypocitraturia, which is less common than hyperoxaluria (8). Citrate is the dissociated anion of citric acid that is utilized due to acidosis (8). This acidosis increases renal citrate reabsorption and reduces excretion in urine (8). It is known to inhibit calcium oxalate and calcium phosphate precipitation through formation of soluble complexes with calcium (8). Prospective studies with non-store formers had decreased urinary citrate, calcium and total urine volume in 45 patients (15), decreased urinary citrate excretion in 21 patients (16) and decreased urine volume and decreased citrate volume in 13 patients (18) after RYGB. A study including 151 patients post RYGB showed that urinary volume (1310 vs. 930mL, p<0.001), citrate (268 vs. 170 mg, p<0.001) and calcium (195 vs. 105 mg, p<0.001) decreased significantly postoperatively (preoperative vs. postoperative, respectively) (19). In a retrospective study among 39 non-stone formers (28 females and 11 males, mean age = 51.2 years), 52% of RYGB patients had urinary citrate value of <370 mg/day vs. 9% of patients with gastric band but both had decreased urine volume (20). Another retrospective study had similar results in 38 patients wherein around 50% had lower urinary citrate levels (22). A previously mentioned study that included 55 patients with follow-up stones, 248 patients without follow-up stones and 20 obese controls with follow-up stones showed that urine citrate was lower in the group with stones vs. the group without stones (p<0.001) (12).

### Infertility and Erectile Function

There is scarce data about the effect of bariatric surgery on fertility and sperm production (6), but rising evidence has shown an association between bariatric surgery and subfertility (10). It has been proposed that excess bodyweight affects sperm production, but it is still not explained if weight loss through operation can reverse this effect (10). Multiple case reports have shown that bariatric surgery affects fertility: six patients (mean age=38 years) with a previous child who underwent RYGB presented with secondary in-fertility/azoospermia after surgery (11). These patients with azoospermia who had testicular biopsy showed spermatogenesis arrest at the spermatogonium stage (11). In another study, three patients had worsened semen parameters during the first year post RYGB such as oligoasthenoteratozoospermia, but none had azoospermia (10). A prospective study on 31 patients (23 RYGB and 8 controls) evaluated seminal parameters six months post-surgery (23). There was a statistically significant increase in gonadotropins (follicle - stimulating hormone and luteinizing hormone), total testosterone, sex-hormone-binding-globulin, calculated free testosterone, increase in semen volume and semen viability and decrease in estradiol (23). There are several explanations for subfertility post-surgery. Undernutrition and disruption of normal pulsatile gonadotrophin-releasing hormone secretion, nutritional deficiencies and release of such liposoluble toxic substances can negatively impact spermatogenesis (10). Other mechanisms include insufficient absorption of nutrients needed for spermatogenesis (11). However, the reversibility of spermatogenesis might indicate that correction of nutritional deficiencies and removal of exposure to toxic substances may be helpful (10).

Many studies have investigated the effect of bariatric surgery on erectile function. A prospective randomized controlled long-term trial compared surgical and non-surgical weight loss impact on erectile function and sexual hormones in morbidly obese men (10 patients and 10 controls) (24). The International Index of Erectile Function (IIEF), total testosterone and free testosterone increased significantly in the intervention group (p=0.0224, p=0.0043 and p=0.0149, respectively) two years after the procedure (24). A prospective study on 32 patients who underwent RYGB and undertook the IIEF showed that these patients had significant increase in total testosterone and sex hormone binding globulin (p<0.001) during the fourth year post operation (25). Similarly, a study targeting 14 males who completed the questionnaire also showed that the male brief sexual function inventory increased after six months from surgery but failed to reach significance (26). A prospective study including 46 patients who underwent sleeve gastrectomy and had semen analysis after a year showed that serum testosterone and sperm concentration increased significantly (p<0.001) in patients with azoospermia (p=0.02) and oligospermia (p=0.001) (27). A prospective study on 51 patients (23 RYGB, 14 overweight and obese controls and 14 lean controls) showed that erectile functions scores were better than those of the obese controls (p=0.015) but lower than those of the lean controls (p=0.028) (28). A study randomized 10 rats into control group and 15 into bypass surgery and showed that the operated group had an increase in the ratio of intracavernosal pressure over mean arterial pressure (p=0.021) which was used to determine erectile function. The authors suggested that glucose homeostasis recovery causes metabolic and biochemical restoration (29). This restoration leads to functional recovery in the corpus cavernosum and results in improved erectile dysfunction (29).

The prevalence of obesity and related morbidities has recently risen across the globe (24). Bariatric surgery has been shown to be favorable in this case (26). However, complications have been observed over the past few years as result of bariatric surgery specifically RGYB (9, 12). The effects of weight loss through bariatric surgery on fertility and erectile dysfunction have not been studied well and results are still controversial. Limitations of the above reviewed studies include low response rate (20, 26) and loss to follow-up and patient burden for collecting tests such as urine specimen and semen samples (20). Some studies did not have randomization (12), while some had very small sample sizes (10, 11, 29) which does not help in stratifying data according to many important factors especially age (26). As for the investigations on sexual behavior to assess the effect of surgery on subfertility, some assessments that were utilized did not include quality of life and relationship satisfaction (25), while others may have had selection bias (26). Another important element to consider is that the underlying mechanism for sexual dysfunction, infertility and nephrolithiasis related to obesity is multifactorial (26). This may lead to higher incidence of stones and decline in sexual function after bariatric surgery. Finally, it is important to note the possibility of relapsing obesity and recurrence of co-morbidities post-surgery (28).

## CONCLUSIONS

Bariatric surgery has proven to be effective and beneficial in the management of obesity (19) and obesity related complications (1, 5, 6). However, the severe decrease in the absorption of nutrients after RYGB may cause short-term and long-term problems such as infertility as depicted by some studies (10, 11). Further elucidation of the pathophysiological mechanism for stone formation and negative effect on fertility and spermatogenesis post-surgery remains an active domain for research (8, 11) in this area.

Urologists should take into account the risk of calcium oxalate stone formation post RYGB in order to provide preventive measures such as increased fluid intake and appropriate dietary measures to protect against renal stone formation (4). A metabolic assessment for nephrolithiasis is suggested for patients who undergo RYGB (19). Additionally, preoperative assessment of patients’ nutritional status and micronutrient supplementation should be taken into account (1). Prospective studies are warranted to confirm the possible benefit of restrictive bariatric procedures on the decreased risk of stone formation in addition to the effect on fertility and erectile function.
